# Where in 1902?

**Published:** 1902-01

**Authors:** 


### EDITORIALS.

### WHERE IN 1902?

MINNESOTA, with her beautiful twin cities of the North, is the last to enter the field for the meeting of the A. V. M. A. in 1902; but, like her able representatives of the profession in all else, she has entered to win, and with vigor and determination that deserve to win she is making a strong plea all along the line to gain this recognition. She has enlisted the officials of the State, the municipal officers of Minneapolis, her Trades League, and a united profession of her own State. Not content with all that this stands for, she is invoking the aid of the profession throughout the West, and, stretching North, she has stirred up her colleagues in Manitoba, and gives fair warning to all the other cities that she is not to be last in the race. Next to Helena, Montana, she offers the greatest attraction as a new field for missionary work, and will equally try in a measure the strength of our growth in live membership. Surely a goodly array of cities—Columbus, Cleveland, Ohio; Chicago, Ill.; St. Louis, Mo.; Kansas City, Mo.; Minneapolis, Minn., and last, but not least, fair Helena, of the Northwest.

